# Estimation of Renal Function Using Unenhanced Computed Tomography in Upper Urinary Tract Stones Patients

**DOI:** 10.3389/fmed.2020.00309

**Published:** 2020-07-03

**Authors:** Jiali Li, Yang Xun, Cong Li, Yunfeng Han, Yaqi Shen, Xuemei Hu, Daoyu Hu, Zheng Liu, Shaogang Wang, Zhen Li

**Affiliations:** ^1^Department of Radiology, Tongji Hospital, Tongji Medical College, Huazhong University of Science and Technology, Wuhan, China; ^2^Department of Urology, Tongji Hospital, Tongji Medical College, Huazhong University of Science and Technology, Wuhan, China; ^3^Department of Radiology and Nuclear Medicine, Tongji Hospital, Tongji Medical College, Huazhong University of Science and Technology, Wuhan, China

**Keywords:** unenhanced CT, differential renal function, residual parenchymal volume, CT texture analysis, upper urinary tract stones

## Abstract

**Objectives:** The aim of this study was to determine whether unenhanced computed tomography (CT) imaging can estimate differential renal function (DRF) in patients with chronic unilateral obstructive upper urinary tract stones.

**Materials and Methods:** This was a single-center retrospective study of 76 patients. All the patients underwent unenhanced CT and nuclear renography (RG) at an interval of 4 to 6 weeks due to chronic unilateral obstructive urinary stones. Renal CT measurements (RCMs), including residual parenchymal volume (RPV) and volumetric CT texture analysis parameters, were obtained through a semiautomatic method. Percent RCMs were calculated and compared with renal function determined by RG.

**Results:** The strongest Pearson coefficient between percent RCM and DRF was reflected by RPV (*r* = 0.957, *P* < 0.001). Combinations of RPV and other parameters did not significantly improve the correlation compared with RPV alone (*r* = 0.957 vs. *r* = 0.957, 0.957, 0.887, 0.815, and 0.956 for combination with Hounsfield unit, parenchymal voxel, skewness, kurtosis, and entropy, respectively; all *P* < 0.001). Percent RPV was subsequently introduced into linear regression, and the equation y = −2.66 + 1.07^*^ × (*P* < 0.001) was derived to calculate predicted DRF. No statistically difference was found between predicted DRF using the equation and observed DRF according to RG (*P* = 0.959).

**Conclusion:** Unenhanced CT imaging can estimate DRF in patients with chronic unilateral obstructive upper urinary tract stones, and RG might not be necessary as a conventional method in clinical.

## Introduction

Urolithiasis is the most common disease that encountered in urology departments, and affects 1–20% of the adult population. In developed countries, such as Canada, Sweden and the United States, the prevalence of urolithiasis is greater than 10% (1). In developing countries, take China for example, kidney stones affect approximately one in 17 adults, corresponding to the prevalence of 5.8–6.5% in men and 5.1% in women (2). Owing to the lack of periodic physical examination, a large number of patients experience acute or chronic obstruction and renal failure. Estimation of differential renal function (DRF), which reflects the contribution of a single kidney to overall renal function, of the obstructed kidney is vital to decide whether it is worth saving. A cut-off value of 15% split DRF is commonly used by urologists when counseling patients to undergo lithotripsy vs. nephrectomy (3).

Nuclear renography (RG) is seen as the standard imaging modality for evaluating differential renal function. However, its clinical applications are limited due to some disadvantages (4, 5), including exposure to radiation, operator dependence, high costs, and prolonged examination time. In contrast, wide popularity and short acquisition time of unenhanced computed tomography (CT) make it a first-line examination method for imaging patients with ureteral stones (6, 7). Hence, exploring the relationship between renal CT measurements (RCM) and DRF has been an active area. Nevertheless, the results of previous research may not be applicable to the populations with upper urinary tract stones, given that previous studies primarily focused on patients with ureteropelvic junction obstruction (UPJO) or included heterogeneous population with a wide range of causes of stones (8, 9). Furthermore, some studies may exposed patients to the risk for contrast nephropathy due to the use of iodinated contrast medium (10, 11). Therefore, the research that only focused on patients with upper urinary tract stones and based on unenhanced CT is valuable and urgently needed.

A major emerging trend in medical imaging research is CT texture analysis (CTTA), which is a novel technique used to assess internal structural heterogeneity by processing existing CT images (12). To our knowledge, few studies exploring the relevance of CTTA and DRF have been published. In the present study, residual parenchymal volume (RPV) and CTTA parameters were obtained very readily and accurately by common software and techniques.

Therefore, this study, which focused on patients with chronic unilateral obstructive upper urinary tract stones, first aimed to correlate percent RPV measured by unenhanced CT with DRF estimated by RG, and second aimed to explore applications of volumetric CTTA in estimating DRF.

## Materials and Methods

### Patients

This retrospective study was conducted under the approval of the Ethics Committee of Tongji Medical College, Huazhong University of Science and Technology (2019S1035) and the informed consent was waived. Data from 112 patients with upper urinary tract stones, who underwent unenhanced multi-detector CT (MDCT) and nuclear RG at an interval of 4–6 weeks between April 2016 and May 2018, were reviewed. Inclusion criteria were as follows: (a) the age was more than 18 years; (b) patients had chronic unilateral obstructive upper urinary tract stones but with a normal contralateral kidney; (c) the stone history was more than 2 months. Exclusion criteria were as follows: (a) patents with a solitary kidney; (b) males with a serum creatinine level > 104 μmoI/L, females with a serum creatinine level > 84 μmoI/L; (c) patients with acute obstruction which were found on contrast-enhanced CT; (d) patents with serious urinary infection; (e) patents with obstruction due to ureteral stricture or ureteropelvic junction obstruction (UPJO). According to the inclusion and exclusion criteria, 76 patients were ultimately included in this study.

### Unenhanced CT

All patients underwent abdominal and pelvic CT examinations (Discovery CT 750, GE Medical Systems, USA; or Aquilion One, Toshiba, Japan). The imaging parameters were as follows: slice thickness, 0.625 mm; pitch, 0.984; gantry rotation time, 0.5 s; tube voltage, 120 kV; and automatic tube current modulation, 100–200 mA. A 5-mm interval was used for CT image reconstruction.

**Figure 1:**
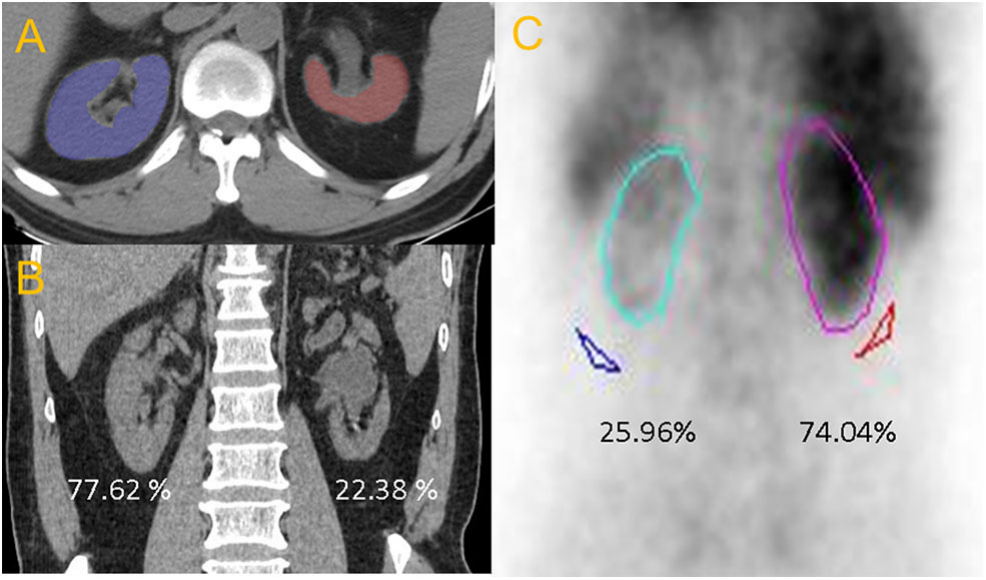
**(A)** Axial non-enhanced CT image. **(B)** The percentage of CT renal volume for right and left kidney were 77.62 and 22.38%, respectively. **(C)** The differential renal function from RG of right and left kidney were 74.04 and 25.96%, respectively.

All patients' CT Digital Imaging and Communications in Medicine (DICOM) images were transferred to a dedicated image analysis workstation equipped with an open source software (Fire Voxel, New York University, NY, USA), and then processed by an abdominal radiologist in a double-blinded manner. First, renal parenchyma of two sides were drawn on the superior and inferior layers of the axial kidney image. The software automatically filled the entire kidney according to the Hounsfield unit (HU) threshold to obtain a volume of interest (VOI), including the renal cortex and medulla. Second, the VOI was magnified, and the edges were manually modified to ensure that all functional renal parenchyma was contained while hydronephrosis, calculi, and cysts were avoided (Figure 1). Third, RCMs (morphological and CTTA parameters) were automatically calculated based on the final VOI, including RPV, HU, parenchymal voxel, skewness, kurtosis, and entropy.

### Nuclear RG

RG values were recorded based on the reports compiled by a professional radiologist. All DRF on RG data were blindly obtained from an observer and processed using the Discovery NM670 SPECT/CT platform (GE Healthcare, USA).

### RCM Analysis

RCMs were evaluated for their power of correlation to DRF. The percent RCM were calculated using the general format “100^*^ [Left RCM / (Left RCM + Right RCM)]” as previously reported (8, 13). All percent RCMs used the left kidney, regardless of whether it was obstructed. The contralateral side can always be determined by assigning the remaining percentage because the two sides total 100. The percent RCM were then compared with the DRF, as determined by RG.

### Statistical Analysis

Continuous variables were expressed as mean ± standard variation. Pearson's correlation coefficient and linear regression were used to evaluate correlations between percent RCM and DRF on RG. A *p* value < 0.05 was considered to indicate a statistically significant difference. SPSS version 24 (IBM Corporation, Armonk, NY, USA) was used to perform the statistical analyses. The difference in correlation coefficient between men and women was calculated using MedCalc (version 12.7, Mariakerke, Belgium).

## Results

A total of 31 men and 45 women, who underwent unenhanced CT and nuclear RG, were included in the analysis. Baseline patient characteristics were summarized in Table 1. The mean age of the patients was 51.14 ± 10.61 years, and the mean serum creatinine level was 76.05 ± 15.4 μmoI/L. There were 45 cases of right-sided stones and 31 cases of left-sided stones. Eight patients had hypertension and five patients had diabetes, but all of them were well controlled. Ureteroscopic lithotripsy was performed in 16 cases, and percutaneous nephrolithotomy was performed in 33 cases, the remaining 27 patients underwent laparoscopic nephrectomy.

**Table 1:** Baseline cohort characteristics, *n* = 76.

**Characteristic**	
Age in years–mean ± SD	51.14 ± 10.61
Serum creatinine–mean ± SD	76.05 ± 15.4
Gender–no. (%)	
Male	31 (40.8%)
Female	45 (59.2%)
Preoperative drainage–no. (%)	
Yes	13 (17.1%)
No	63 (82.9%)
Drainage type–no. (%)	
Drainage type	6 (46.2%)
Double J ureteral stents	4 (30.8%)
Drainage type + double J ureteral stents	3 (23.0%)
Stone side–no. (%)	
Right	45 (59.2%)
Left	31 (40.8%)
Hypertension–no. (%)	
Yes	8 (10.5%)
No	68 (80.5%)
Diabetes–no. (%)	
Yes	5 (6.6%)
No	71 (93.4%)
Operation for stone–no. (%)	
Ureteroscopic lithotripsy	16 (21.1%)
Percutaneous nephrolithotomy	33 (43.4%)
Laparoscopic nephrectomy	27 (35.5%)

*SD, standard deviation*.

The percent RPV, HU, parenchymal voxel, skewness, kurtosis, and entropy were separately evaluated for their strength of correlation to DRF on RG. In addition, multiple combinations of these RCMs were also used to evaluate the correlation with DRF. For each RCM, a Pearson's correlation coefficient between the percent RCM and the DRF was calculated (Table 2). The strongest correlation between RCM and DRF was achieved with RPV (*r* = 0.957, *P* < 0.001). However, RPV multiplied by HU, and parenchymal voxel multiplied by HU, also achieved the same correlation (*r* = 0.957, *P* < 0.001). Moreover, there was a relatively high correlation between the percent RPV multiplied by entropy and DRF (*r* = 0.956, *P* < 0.001). The results of gender-based subgroup analysis were shown in Table 3. Whether in men or women, RPV has still achieved the strongest correlation between RCM and DRF (*r* = 0.962 for female, *r* = 0.950 for male). Although the correlation coefficient of women is slightly larger than that of men, the difference is not statistically significant (*P* = 0.565).

**Table 2:** Evaluating differential CT measurements and their correlation to DRF on RG.

**Measurement/Calculation**	**Pearson correlation**	***p*-value**
CT Texture:		
Skewness^*^	0.282	0.013
Kurtosis^**^	0.297	0.009
Entrophy	−0.22	0.849
HU	0.198	0.086
Parenchymal Voxel^***^	0.956	<0.001
Volume		
Parenchymal Volume^***^	0.957	<0.001
Combinations		
Parenchymal Volume × HU^***^	0.957	<0.001
Parenchymal Volume × skewness^***^	0.887	<0.001
Parenchymal Volume × Kurtosis^***^	0.815	<0.001
Parenchymal Volume × Entropy^***^	0.956	<0.001
Parenchymal Voxel x HU^***^	0.957	<0.001
Parenchymal Volume × Parenchymal Voxel x HU^***^	0.951	<0.001

*Parenchyma defined as both renal cortex and medulla. HU, Hounsfield Units*.

* *P < 0.05*,

** *P < 0.01*,

*** *P < 0.001*.

**Table 3:** Gender subgroup analysis of the correlation between differential CT measurements and DRF on RG.

	**Male (*****n*** **=** **31)**		**Female (*****n*** **=** **45)**	
**Measurement/Calculation**	**Pearson correlation**	***p*-value**	**Pearson correlation**	***p*-value**
HU	0.673	<0.001	0.249	0.099
Parenchymal Voxel	0.948	<0.001	0.962	<0.001
Parenchymal Volume	0.950	<0.001	0.962	<0.001
Parenchymal Volume × HU	0.950	<0.001	0.962	<0.001
Parenchymal Voxel × HU	0.949	<0.001	0.962	<0.001
Parenchymal Volume × Parenchymal Voxel × HU	0.941	<0.001	0.958	<0.001

*Parenchyma defined as both renal cortex and medulla. HU, Hounsfield Units*.

**Figure 2:**
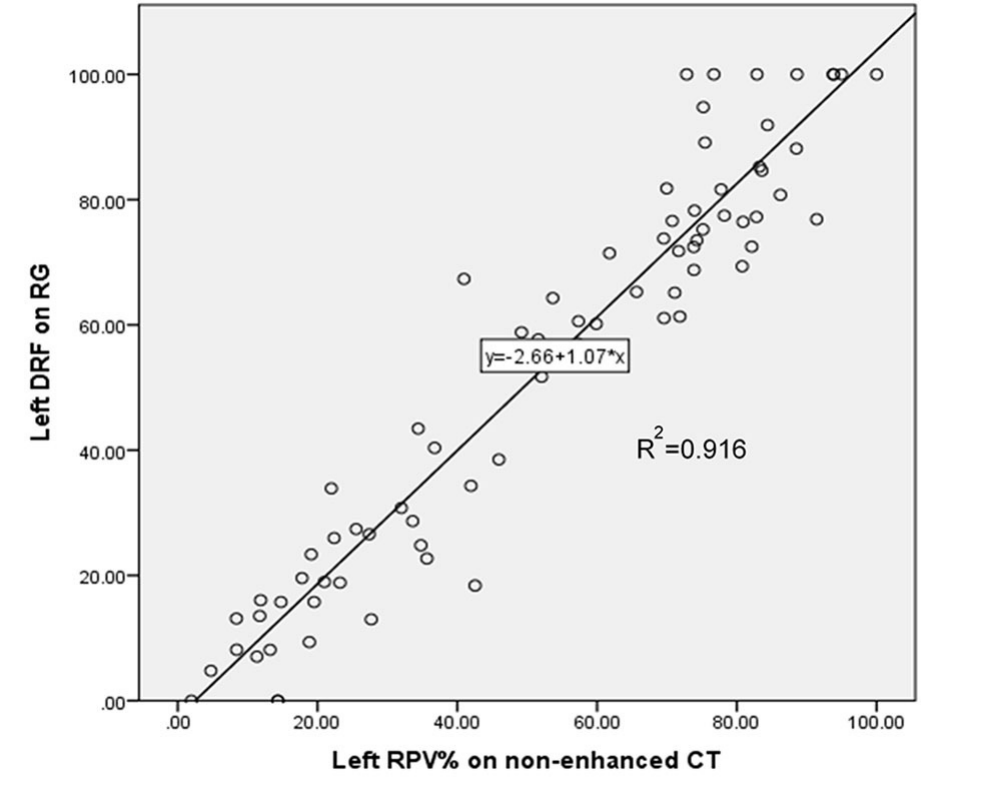
Linear regression of unenhanced computerized tomography (CT) percent left residual parenchymal volume (RPV) estimating left differential renal function (DRF) on nuclear renography (RG).

Linear regression produced an equation for estimating DRF: y = −2.66+1.07^*^ x” (*P* < 0.001), in which “x” is the left percent RPV, and “y” is the equation-estimated DRF (Figure 2). The numerical values comparing the percent RPV, equation-estimated DRF, and actual reported DRF from RG were provided in Supplementary Table S1. No statistically significant difference was observed between equation-estimated DRF and actual reported DRF (*P* = 0.959).

## Discussion

Chronic urinary tract obstruction caused by stone often leads to renal impairment of the affected unit, and DRF of the obstructed kidney can impact the surgical decision. Urologists usually determine split DRF by nuclear RG. However, high cost of nuclear RG precludes some hospitals from performing this examination. Therefore, given increases in morbidity of urolithiasis (1), the need of using unenhanced CT (basic examination) to avoid nuclear RG is also on the rise. In this preliminary study, the percent RPV from unenhanced CT demonstrated a strong correlation with DRF from RG (*r* = 0.957). Moreover, DRF was easily and precisely evaluated using the equation from unenhanced CT.

In 2016, Jacob et al. (8) investigated 49 patients with UPJO and reported a strong correlation between percent cortical area multiplied by HU on CT and DRF of RG. Studies in 2014 by Hamed et al. (10) and in 2012 by Sarma et al. (14), including 42 and 21 patients, respectively, found that percent RPV on CT strongly correlated with DRF using 99mTc-DTPA renal scan. As mentioned above, multiple studies have indicated that DRF can be adequately assessed using percent RCM, and previous results were compatible with those of this study. However, enhanced CT was used most frequently in previous studies. Despite the strong correlation, enhanced CT as a tool for assessing DRF is not without limitations, primarily because the exposure to contrast medium adversely affects renal function (15). Enhanced CT is prohibited in patients with worsening renal function (16) or allergy to contrast media. Furthermore, some studies (8, 17, 18) have applied HU to calculate CT-based DRF; however, it is difficult to standardize the data because tissue attenuation derived from enhanced CT is affected by a variety of factors (19, 20), including contrast medium type, injection concentration and rate, and image acquisition time. However, these disadvantages do not apply in unenhanced CT. Hence, a reasonable assumption could be drawn that the results of this study may be more easily adopted and validated by other medical centers, and easier to be generalized into clinical practice.

In 2010 Morrisroe et al. (13) and in 2015 Martinez-Suarez et al. (21), compared percent RPV on unenhanced CT with DRF on nuclear renal scan. The common limitation of their research, however, was the rather small sample size (10 and 19 patients, respectively). A relatively larger sample (*n* = 72) was studied by Feder et al. (9), and their results indicated a strong correlation between percent renal parenchymal area from unenhanced CT and DRF from renal scintigraphy, similar to the results of the present study (Pearson's *r* = 0.967 vs. *r* = 0.957). However, the 72 patients had a wide range of disease types (and included kidney donors). In contrast, the inclusion criteria for this study were strictly controlled (only including patients with chronic unilateral obstruction upper urinary tract stones). Therefore, the statistical power of the results from the present study may be stronger, especially in patients with upper urinary tract stones. In addition to the classic morphological indicator (RPV), relatively novel CTTA parameters were also introduced to this study.

Numerous studies have demonstrated that CTTA can reflect heterogeneity in the microstructure (12, 22, 23). Given that it is not unusual for chronic renal obstruction to undergo the changes in kidney texture during functional impairment, this study sought to ascertain whether CTTA could accurately reflect DRF. This research demonstrated a strong correlation between percent CTTA parameters multiplied by RPV and DRF. Although the combinations did not significantly improve the correlation compared with individual RPV, this result still reflected a certain value. First, this study and its findings may provide a reference for researchers who are committed to studying the relationship between renal function and CTTA. Second, it could be reasonably inferred that CTTA parameters may be more helpful in situations for which RPV does not accurately reflect renal function. For example, the expanded interstitial spaces caused by active infection or acute obstruction can lead to enlarged renal volume (13), further reducing the correlation between renal volume and function. Whereas microenvironmental changes may be recognized by CTTA parameters, the use of CTTA to detect acute myocardial infarction supports this hypothesis (24, 25). Hence, the contribution of CTTA may exceed the renal volume in such situations, although further verification is needed.

Although this research verified a correlation between CT measurements and DRF, it helped only a little in clinical practice; therefore, this study further introduced RPV into the equation from linear regression to obtain a CT equation-estimated DRF. Importantly, the difference between equation-estimated DRF and reported DRF according to RG did not fulfill formal statistical criteria. Consequently, this equation may be used as a method to prospectively screen patients for further validation, at least in this single medical center.

There were several limitations to this investigation. First, although previous studies (9, 13) have reported that the correlations were weakened in patients with worse renal function, this study did not perform subgroup analysis based on renal function due to the relatively small sample size. Interestingly, the correlation between percent RPV and DRF in this study may be stronger theoretically if patients with a split renal function of zero are excluded. In future, larger-sample studies, subgroup analysis based on renal function will be considered more cautiously and comprehensively. Second, postoperative renal function was not analyzed in this study due to the lack of follow-up data. Although this is not inconsistent with the purpose of this study (focusing on preoperative patients), it is necessary to include postoperative renal function assessment in further studies. Third, reproducibility was not evaluated, given that previous studies (26, 27) have demonstrated excellent intra-class correlation coefficient (ICC) values for RCM (>0.9). If the ICC test was performed, the results of this study using three-dimensional volume software should be at least as good as those of previous studies.

## Conclusion

In conclusion, for patients with chronic unilateral obstructive upper urinary tract stones, unenhanced CT enabled preoperative DRF to be rapidly and accurately estimated, and the role of RPV is more important than CTTA parameters. Measuring RPV on unenhanced CT images may assist urologists in determining optimal treatment strategies. Probably in most chronic unilateral obstructive upper urinary tract stones patients, measuring RPV on unenhanced CT may be sufficient to evaluate renal function, and RG may not be necessary as a conventional method in clinical.

## Data Availability Statement

All datasets generated for this study are included in the article/Supplementary Material.

## Ethics Statement

The studies involving human participants were reviewed and approved by Ethics Committee in Tongji Medical College. Written informed consent for participation was not required for this study in accordance with the national legislation and the institutional requirements.

## Author Contributions

JL and YX: writing original draft and acquisition of data. CL and YH: data analysis and interpretation. YS, XH, and DH: literature research. ZLiu, ZLi, and SW: revising article and study supervision. All authors contributed to the article and approved the submitted version.

## Conflict of Interest

The authors declare that the research was conducted in the absence of any commercial or financial relationships that could be construed as a potential conflict of interest.
