# Supplementary material for: Estimation of Renal Function Using Unenhanced Computed Tomography in Upper Urinary Tract Stones Patients
Source: Front Med (Lausanne). 2020 Jul 3;7:309. doi: 10.3389/fmed.2020.00309 (PMC7347744; doi:10.3389/fmed.2020.00309)
Supplement: Supplementary file 1 [file Table_1.docx]

Supplementary Table S1. Comparison among CT left percent residual parenchymal volume (RPV), equation-estimated left differential renal function (DRF), and renogram (RG) left DRF

| Patients | percent RPV^a^ | Equation-estimated DRF^b^ | RG DRF^c^ |
| --- | --- | --- | --- |
| 1 | 11.86 | 10.03 | 16.05 |
| 2 | 82.80 | 85.94 | 77.24 |
| 3 | 75.20 | 77.81 | 94.77 |
| 4 | 1.95 | -0.58 | 0.00 |
| 5 | 13.23 | 11.49 | 8.12 |
| 6 | 27.41 | 26.67 | 26.58 |
| 7 | 51.60 | 52.55 | 57.70 |
| 8 | 80.89 | 83.89 | 76.45 |
| 9 | 71.67 | 74.02 | 71.79 |
| 10 | 71.85 | 74.22 | 61.33 |
| 11 | 72.83 | 75.26 | 100.00 |
| 12 | 77.75 | 80.54 | 81.63 |
| 13 | 4.74 | 2.41 | 4.78 |
| 14 | 94.98 | 98.97 | 100.00 |
| 15 | 75.45 | 78.07 | 89.11 |
| 16 | 19.10 | 17.78 | 23.35 |
| 17 | 91.42 | 95.16 | 76.87 |
| 18 | 83.57 | 86.76 | 84.63 |
| 19 | 34.79 | 34.56 | 24.81 |
| 20 | 73.87 | 76.38 | 68.77 |
| 21 | 14.33 | 12.67 | 0.00 |
| 22 | 8.40 | 6.32 | 13.12 |
| 23 | 59.88 | 61.41 | 60.16 |
| 24 | 73.83 | 76.34 | 72.45 |
| 25 | 41.96 | 42.23 | 34.30 |
| 26 | 8.43 | 6.36 | 8.13 |
| 27 | 34.41 | 34.16 | 43.45 |
| 28 | 21.98 | 20.86 | 33.89 |
| 29 | 78.21 | 81.03 | 77.45 |
| 30 | 25.53 | 24.66 | 27.41 |
| 31 | 88.52 | 92.05 | 88.15 |
| 32 | 32.00 | 31.58 | 30.77 |
| 33 | 14.32 | 12.66 | 0.00 |
| 34 | 82.88 | 86.02 | 100.00 |
| 35 | 52.37 | 53.38 | 56.70 |
| 36 | 71.11 | 73.43 | 65.14 |
| 37 | 93.84 | 97.75 | 100.00 |
| 38 | 42.54 | 42.86 | 18.38 |
| 39 | 57.17 | 58.51 | 57.05 |
| 40 | 74.28 | 76.82 | 73.51 |
| 41 | 18.84 | 17.50 | 9.35 |
| 42 | 82.13 | 85.22 | 72.49 |
| 43 | 69.96 | 72.19 | 81.79 |
| 44 | 93.79 | 97.70 | 100.00 |
| 45 | 86.24 | 89.61 | 80.75 |
| 46 | 88.62 | 92.16 | 100.00 |
| 47 | 33.62 | 33.31 | 28.67 |
| 48 | 11.74 | 9.90 | 13.52 |
| 49 | 75.15 | 77.75 | 75.25 |
| 50 | 69.54 | 71.74 | 73.79 |
| 51 | 57.34 | 58.69 | 60.59 |
| 52 | 22.38 | 21.28 | 25.96 |
| 53 | 70.77 | 73.06 | 76.61 |
| 54 | 35.65 | 35.48 | 22.70 |
| 55 | 61.78 | 63.44 | 71.45 |
| 56 | 65.64 | 67.58 | 65.28 |
| 57 | 40.96 | 41.17 | 67.33 |
| 58 | 73.93 | 76.45 | 78.26 |
| 59 | 19.51 | 18.22 | 15.75 |
| 60 | 23.22 | 22.19 | 18.84 |
| 61 | 27.69 | 26.97 | 12.98 |
| 62 | 83.24 | 86.41 | 85.26 |
| 63 | 20.98 | 19.79 | 18.97 |
| 64 | 53.66 | 54.76 | 64.29 |
| 65 | 14.76 | 13.14 | 15.75 |
| 66 | 69.58 | 71.79 | 61.09 |
| 67 | 100.00 | 104.34 | 100.00 |
| 68 | 17.78 | 16.36 | 19.58 |
| 69 | 11.33 | 9.46 | 7.03 |
| 70 | 80.77 | 83.76 | 69.36 |
| 71 | 76.71 | 79.42 | 100.00 |
| 72 | 45.95 | 46.50 | 38.51 |
| 73 | 49.19 | 49.97 | 58.82 |
| 74 | 36.75 | 36.67 | 40.37 |
| 75 | 52.06 | 53.04 | 51.73 |
| 76 | 84.39 | 87.63 | 91.90 |

^a^Left percent RPV = 100*(Left RPV / [Left RPV + Right RPV]).

^b^Equation-estimated left DRF =-2.66+1.07*x, where 'x' is the value of left percent RPV.

^c^The value of the left kidney's DRF on renogram.
